# Indigenous biosystematics of yams (*Dioscorea* spp.) in Southwest Ethiopia: folk taxonomy, ethnolinguistic analysis, and folk descriptors

**DOI:** 10.1186/s13002-020-00427-8

**Published:** 2021-01-02

**Authors:** Tsegaye Babege Worojie, Bizuayehu Tesfaye Asfaw, Wendawek Abebe Mengesha

**Affiliations:** 1 Department of Horticulture, Mizan-Tepi University, P. O. Box 260, Mizan-Teferi, Ethiopia; 2 School of Plant and Horticultural Science, Hawassa University, P.O. Box 05, Hawassa, Ethiopia; 3 Department of Biology, Hawassa University, P. O. Box 05, Hawassa, Ethiopia

**Keywords:** Indigenous biosystematics, *Dioscorea* species, Folk taxa, Yam varieties

## Abstract

**Background:**

In Southwest Ethiopia, various plant species are coexisting in wild and cultivated forms. This provides an ideal setting for studying folk biosystematics of neglected species. One of such species is the *Dioscorea* species, in which we studied to assess the commonly applied folk wisdom of identifying, naming, and classifying yams by Sheko and Bench farmers.

**Methods:**

This study was conducted in Bench-Maji and Sheka Zones using 272 farmers. Data on the lists of local names and system of folk taxonomy; the inherent logic, etymons, and consistency of names; and the folk descriptors and other criteria involved in taxonomy were collected. Data were collected by establishing participatory research appraisal tools, i.e., informant interviews and researcher direct observation.

**Results:**

The result suggests that there exists a well-developed folk taxonomic system in Sheko and Bench. This is evident in the recognition of four distinct folk ranks: sub-variety, variety, supra-variety, and folk generic. Taxa assigned to each ranks have distinct features that mark them as members of a separate categories. Farmers over-differentiate 58 individual taxa at the intraspecific levels. Of these, 37 represented varietal taxa while the rest are sub-varietal taxa. Structurally, over 78% of the varieties are labeled with unitary names while all the sub-varieties consisted of binomial names. Farmers used a total of 26 characters and 74 character states for identifying the different taxa. More than 84% of these refer to aspects of plant characteristics. Tuber characters played a key role in the local identification of varietal and sub-varietal taxa while contexts and gender played a key role in the recognition of supra-variety groups.

**Conclusions:**

This study documented a great wealth of knowledge on indigenous biosystematics of yams, constitutes an essential step towards setting development priorities aimed at in situ conservation. The study clearly demonstrated the value of folk biosystematics for assessing the actual extent and spatial dynamics of yam diversity in traditional farming*.*

## Background

Root and tuber crops such as potato, sweet potato, enset, cassava, and yam play an enormous role in feeding the world. They are among the most adaptable staples addressing food security for millions of peoples globally and are nutritionally rich staples that contribute towards the dietary demands of the society. Thus, they serve as an important safety net against starvation. Many of these crops are grown primarily for subsistence, under traditional farming systems, which still represent much of world agriculture. These agro-ecosystems retain a great diversity potential for future use, yet studies on the evolution and conservation needs of these crops are few. Evolution under domestication is affected by the management of folk cultivars, mainly when humans act as agents of selection and is thus said to be linked with the knowledge of indigenous biosystematics of crop plants [1–3]. Yam is one of such crops which needs high conservation concern.

The word yam is applied only to members of the genus *Dioscorea* that belong to the family Dioscoreaceae classified under the monocotyledons [4]. *Dioscorea* is one of the largest genera in this family comprising over 600 species [5]. It is a pantropical genus, and different species have been independently domesticated in three separate continents [6]. The genus *Dioscorea* was first described by Linnaeus in 1753, when he considered three species, but its division into botanical sections was much more recent. In 1924, Knuth established the prevailing systematic and then, *Dioscorea* is divided into 5 sections: Enantiophyllum, Opsophyton, Macrogynodium, Combilium, and Lasiophyton. Of these, the section Enantiophyllum is the largest in terms of number of species and includes all the species with a rightward stem twining direction [7]. Of the important yam species, *D. alata* and *D. cayenensis* complex belong to this section [5].

Yams in Ethiopia are hardly known to the scientific community, and in fact, the country is generally regarded to as an isolated center of yam belt [8]. Yet, several yam species might have their origin in Ethiopia and are one of those crops with wild relatives in the country [9, 10]. Miege and Sebsebe [11] reported that the genus *Dioscorea* has 11 species in Ethiopia. Regarding the intra-specific diversity of yam in Ethiopia, at least 134 non-synonymous landraces have been reported by previous works ([12–15], Tsegaye B, Bizuayehu T, Wendawek A: Diversity, Distribution and Farmers Management of Yams (Dioscorea spp.) in Southwest Ethiopia, submitted). Over 97% of these landraces belong to the section Enantiophyllum, and within this section, high degree of polymorphism has been identified in the *D. cayenensis* complex. Though this species complex shows a very wide range of variation, it has not been studied throughout its range.

This paper presents the indigenous biosystematics of yams. Indigenous biosystematics can be defined as the commonly applied and recognized folk wisdom of identifying, classifying, naming, and relating living organisms as practiced by a particular ethnic group [16]. Scientific studies of indigenous biosystematics seek to unravel the classification of folk ranks and taxa, the morphological character states applied in classification and native nomenclatures. In earlier works, the term folk taxonomy is more widely used [17–19]. Though it is a more widely used term, its use in literature may relate to different components of taxonomy ranging from a mere list of local names to a hierarchical system of taxonomy [16]. Its common use does not necessarily address how particular cultures classify living organisms. Moreover, a basic linguistic analysis, i.e., questions concerning the inherent logic and consistency of folk names, are not always considered as part of folk taxonomy. Here, folk taxonomy is considered as one component of folk biosystematics, which involves a broader set of subsystems.

Studying indigenous biosystematics is thus essential at least for two reasons. First and more notably, it provides a better understanding to the nature and extent of diversity and how this diversity is perceived and valued by farmers. Second, by detailing the inherent subsystems of indigenous biosystematics, an insight to its relation with the taxa recognized in the domain of formal science can be obtained. The insight that this provides is valuable for the improvement and conservation of yam in Ethiopia. Finally, it is of utmost importance because it is not only the unit of diversity that they recognize but also the unit of how they actually manage and conserve.

The far Southwest Ethiopians maintain large numbers of intraspecific yam diversity [12, 14]. Each of these is perceived as distinct and given a separate name. But, studies on how farmers identify, name, and classify yams are poorly studied. Accounts on the consistency of folk names and their importance as part of indigenous biosystematics of yams is scarce. Formal descriptors for yam had already been developed early in the nineteenth and twentieth century [20, 21] and have been improved at several instances [22–24]. Equally important but poorly studied aspect is accounts on the use of folk descriptors. The descriptors recognized by farmers have been little studied. The few attempts made in South and Southwest Ethiopia [12, 25] have focused only on folk taxonomy and have not covered the multiple dimensions of folk biosystematics.

This study aimed to test the following hypotheses. Classification of yam by some Southwest Ethiopian farmers is over-differentiated at the intraspecific level [12]. Knowing that such over-differentiation is rare, and is often related with crops of great importance [26], we hypothesized that this knowledge is widely shared within the community and across locations. Assuming that farmers manage a wide range of descriptors and other criteria for classification [12, 14, 25], we hypothesized that classification is based on a consistent sets of descriptors and can influence practical decisions of maintaining diversity. Considering that some locally recognized taxa are consistent to a considerable extent with the variability obtained in studies using standard markers [25, 27, 28], we assumed that folk taxonomy has a clear biological and functional implication. The present study was thus conducted with the objective (i) to investigate how yam species are named, identified, and classified by Sheko and Bench farmers, (ii) to identify a set of orders of folk ranks recognized by local farmers, and (iii) to assess consistency of folk taxonomy across locations and compare it with the formal taxonomy.

## Methods

### Population size and ethnic compositions

**Fig. 1:**
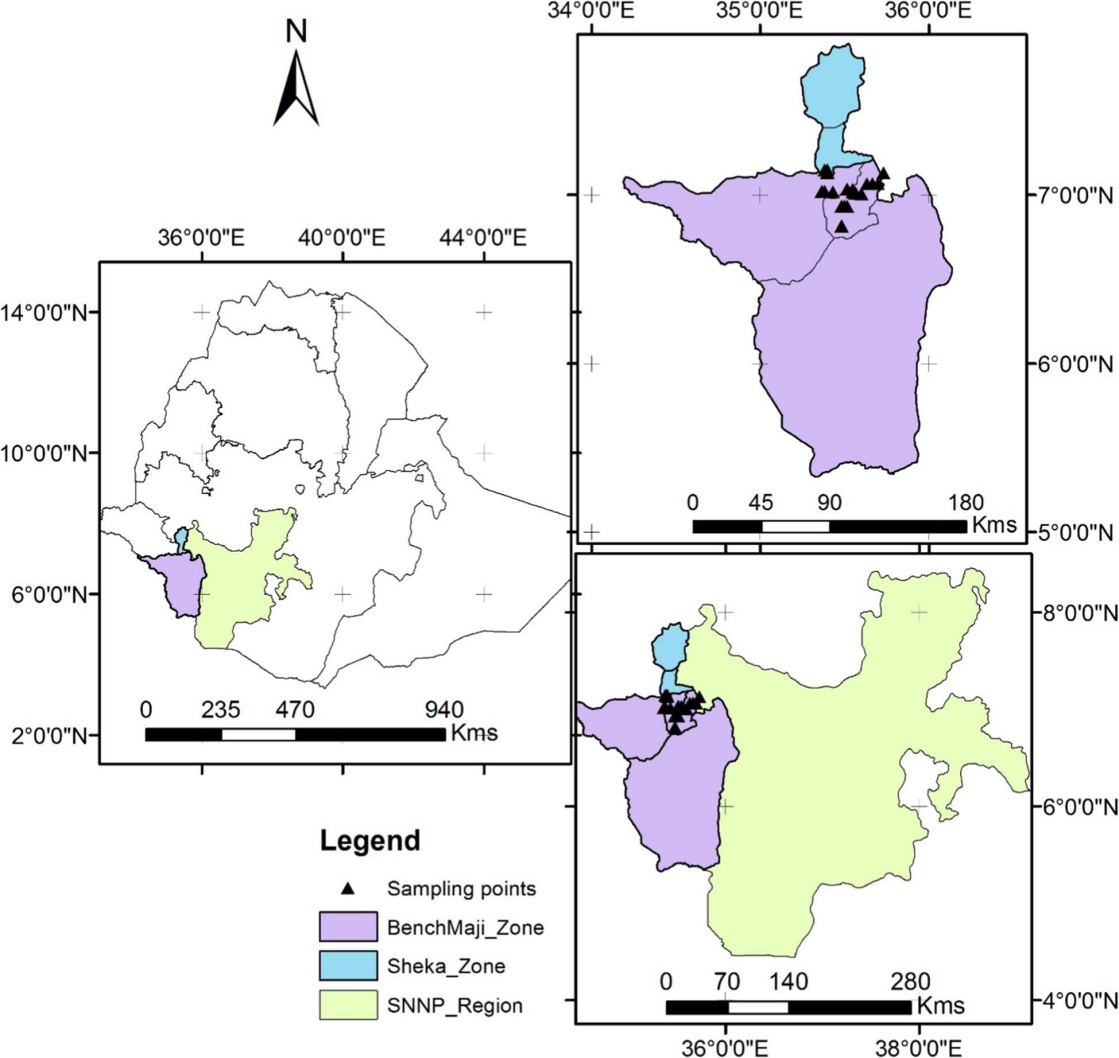
Map of the study areas, indicating the surveyed points in Bench-Maji and Sheka Zones

The study was conducted in Bench-Maji and Sheka Zones of southwest Ethiopia. They are part of the Southern Nations, Nationalities, and Peoples Region (SNNPR) of Ethiopia. The Regions are sub-divided into 13 zones, which are organized into woredas. Kebeles are the smallest administrative units within woredas. Bench-Maji Zone is sub-divided into 11 woredas. Based on the population projection values of 2017, this Zone has a total population of 847,168 of whom 687,212 or 81.12% are rural inhabitants [29]. Bench-Maji Zone is well-known by its multi-ethnic diversity consisting of six main ethnic groups namely Bench (45.11%), Me’enit (21.36%), Surma (3.88%), Dizzi (5.17%), Sheko (4.21%), and Zilmamo (0.22%). The remaining 20.05% comprises a diverse mix of other ethnic groups. Sheka Zone is sub-divided into 3 districts. Based on the population projection values of 2013, this Zone has a total population of 269,243 of whom 196,524 or 72.99% are rural inhabitants [29]. Sheka Zone is also well-known by its multi-ethnic diversity consisting four main ethnic groups namely Shakacho (32.41%), Bench (5.23%), Sheko (4.24%), and Majang (1.73%). The remaining 56.39% of the population comprises a diverse mix of other ethnic groups. Figure 1 presents map of the study area, indicating the surveyed points in Bench-Maji and Sheka Zones.

### Sample and sampling procedure

This study was conducted in five districts of Bench-Maji and Sheka Zones. Yam-growing farmers were purposively considered for this survey. Secondary data regarding the accessibility and culture of yam farming were assessed in Bench-Maji and Sheka Zones. The secondary data obtained from each Zone were used to select yam-growing districts. A total of five yam-growing woredas (districts) were considered in this study. Within these districts, informal survey was conducted to identify yam-farming kebeles. In addition, secondary information regarding the accessibility of yams was assessed in each district. Based on the result of informal survey and secondary data, three kebeles were chosen in each woreda, bringing the total number of sampled households to 272. Informant interviews were adapted from ethnobotanical field inquires as suggested by [30]. Field visits in combination with individual farmer interviews using a standard questionnaire were carried out during the period from December 2016 to November 2017. The data collection procedures employed for recording folk biosystematics is given below.

#### Folk taxonomy

Folk taxonomy was researched with the use of informant interviews, researcher observations, and comparison of farmers’ recognized taxa with formal taxonomy. Folk ranks and taxa were recorded according to the universal scheme proposed by Berlin et al. [26] and Berlin [31]. Accordingly, there are at least five, perhaps six, ethnobiological folk categories which appear to be highly general if not universal in folk biological science. Applying the basic principles proposed by the preceding authors, the categories can be reduced into a set of four general nomenclatural orders as follows:

1. Farmers were asked to free list generic names to all kinds of yams they knew at and above species level. Taxa satisfying these conditions are generic; their labels are generic names.
2. A category called intermediate taxa included the fact that farmers recognized supra-variety categories that are labeled by names. Taxa satisfying these conditions are sub-generic (specific); their labels are supra-variety names.
3. Farmers were asked to free list the names of individual taxa that they perceive as a distinct unit. Taxa satisfying these conditions are varietal; their labels are variety names.
4. Some taxa are marked only by binary lexemes, containing further divisions of a variety. Taxa satisfying these conditions are sub-varietal; their labels are sub-variety names.

#### Ethnolinguistic analysis

A basic linguistic analysis, i.e., questions concerning the inherent logic and consistency of folk names, was researched with the use of farmers’ interview and researcher observations. Individual farmers were asked to free list the local names of yam varieties and sub-varieties they grew or knew. All the folk names were registered, and translation considering meaning, origin, and structures of folk names was made with the use of elderly farmers. The consistency of folk names was assessed with the use of fixed landrace samples. Of the total farms surveyed, landraces that were encountered in more than ten farms were selected as a fixed sample. Accordingly, 15 landraces were identified as fixed samples.

#### Folk descriptors

Folk descriptor was researched based on the farmers’ free listing of individual taxa along with their descriptors, according to informants’ own order of priority without major researcher intervention. Informants were asked to free list the names of (both known and actually grown) individual taxa. For each of the names, farmers were asked how they were able to identify it. All the folk descriptors were registered and supplemented with field observation by the researcher to verify the information gathered.

## Results

### Folk taxonomy

#### Names and naming of yams at and above species level

Farmers have used a broader set of subsystems in naming and classifying yams. Despite their separate taxa in scientific terms, at and above species level, all underground yam types have been merged into Sheko concepts of “*Kachi*”. The Bench, upland Omotic-speaking peoples to the east of Sheko, knew yam by the folk generic name “*Boyye*”. But, Bench still credits the Sheko with having initiated yam culture; some of the Bench farmers knew it by the Sheko generic name, “*Kachi*”. The cultivated *bulbifera* plant has different generic names. In Sheko, it is usually called “*Ama*”, whereas it is called “*Oake*” in Bench. Farmers also provide the generic names for wild yams. The prefixes such as “*Karka*” and “*Shah*” added to the names of wild yams are referring to location names in the wild places (Table 1). Thus, “*Kachi*” is a generic term that is usually used to refer to the underground yam types at the genus level, but not entirely applied to yam species. When farmers were asked to free list all kinds of yams they grew or knew, they linked plants of other species to yams though they are not yam. For instance, cassava (*Manihot esculenta*) is to some extent linked to yams and commonly known as *Enchet-Kachi* (Table 1).

**Table 1:** Sheko and Bench folk classification of yams at and above the species level

Scientific name	References	Context	Folk generic names	
			Sheko	Bench
*Dioscorea alata*	Present study, [10–12]	Cultivated	*Kachi*	*Boyye/Kachi*
*Dioscorea bulbifera*	Present study, [10–12]	Wild	*Karka-Ama*	*Balakay-Oake*
		Cultivated	*Ama*	*Oake*
*Dioscorea cayenensis* complex	Present study, [10–12, 27, 28]	Wild	*Karka-Kachi*	*Shah-boy* (*Karckabat*)
		Cultivated	*Kachi*	*Boyye/Kachi*
*Manihot esculenta*	Present study	Cultivated	*Enchet-kachi*	*Enchet-kachi*

#### Names and naming of sub-variety, variety, and supra-variety

At least two well-recognized species (*D. bulbifera* and *D. alata*) and one species complex (the *D. cayenensis* complex) grow in the study area*.* The far Southwest Ethiopians classify these species into at least 37 varietal taxa. Some varieties are further composed of a number of subordinate units (sub-varieties), each of which are reported to differ from all the others at least in one feature. In our study, ten varieties consisted of 21 sub-varieties, bringing the total numbers of locally recognized individual taxa to 58 (Table 2). Of these, 34 represented actually grown landraces [16]. Besides these, six additional landraces were found in farms of unsampled households while the rest reported verbally. Yams in the lowest taxonomic levels, i.e., at varietal and sub-varietal ranks, are further grouped into distinct supra variety categories. These categories are labeled by names and usually group several varieties and sub-varieties together by a single criterion; they thus constitute intermediate ranking. Table 3 presents a description of nine supra-variety groups that are common in Sheko and its environs.

**Table 2:** Lists of variety and sub-variety names and the underlying basis of the classification

List of local names		References	Contexts	Structures	Meaning of names or the underlying basis of classification /identification
**I.** ***D. cayenensis*** **complex**					
1	*Karka-kachi*	Present study, [12, 15]	W	PU	“*Karka*” means forest in Sheko dialect, and its name is said to be derived from the wild location. Whole tuber of *Karka-Kachi* is dark purple and bitter in taste. If tuber has bright orange color at stem junction, it is red (*submseb*); if it has dark grayish purple pigment at proximal end, it is black (*tsaa’nseb*), and if it is unpigmented, it is white (*ga’nseb*).
	*Karka-kachi submseb*	Present study, [12]		PB	
	*Karka-kachi tsaa’nseb*				
	*Karka-kachi ga’nseb*				
2	*Shah-boy, Karckabat*	Present study, [14]	W	PU	“*Shah*” means forest in Bench dialect; its name thus refers to the area name in a wild place. Its tuber has pale purple color with few pale orange colors at proximal end.
3	*Upfa*^a^	[12]	W	SU	Name translation is not given. Yet, its tuber has patches of purple pigment at proximal end and white elsewhere
4	*Karka-Kachi*	Present study, [12]	WT	PU	Tuber of this variety is generally pale purple. If it has dark purple pigment at stem junction, it is black (*tsaa’nseb*); if it is unpigmented, it is white (*ga’nse*b); if it has patches of orange color at proximal end and white elsewhere, it is spotted (*guignseb*).
	*Karka-kachi tsaa’nseb*			PB	
	*Karka-Kachi ga’nseb*				
	*Karka-Kachi guignseb*				
5	*Yasind*	Present study, [14]	WT	SU	Tubers of *Yasind* varieties are very similar in size and shape. But, if its tuber has purple pigment at proximal end, it is black (*Tsid*); if it has patches of purple and orange color at proximal end and white elsewhere, it is white (*Dal*)
	*Tsid yasind*			SB	
	*Dal yasind*				
6	*Kaibab/Baidai-kachi*	Present study, [12]	RT	PU	*Kaibab* is the area name in northeast Sheko. *Baidai* is said to come from a *Kaibab* area. Whole tuber is purple with white, but if it is grayish purple at proximal end, it is black (*tsaa’nseb*); if it is unpigmented, it is white (*ga’nseb*).
	*Kaibab-kachi tsaa’nseb*			PB	
	*Kaibab-kachi ga’nseb*				
7	*Chabsha*	Present study, [14, 15]	RT	SU	Tubers of *Chabsha* varieties are similar in size and shape, but *Tsid Chabsha* has pale purple proximal end and pale gray stems, while *Dal Chabsha* does not.
	*Tsid Chabsha*	Present study, [14]		SB	
	*Dal Chabsha*				
8	*Tolubab*	Present study, [14]	RT	SU	Name means “tubers with a bitter taste”. Tuber has patches of pale purple color at proximal end and white elsewhere
9	*Torbay*	Present study, [12, 15]	RT	SU	Name means “tubers with a bad taste”. Tuber has patches of pale orange color at proximal end and white elsewhere
10	*Beri*	Present study, [12, 14]	LT	SU	Name means “lowland desert” wherein it is said to come from lowland desert areas. The tuber and basal stem edges of this variety have pale purple pigment.
11	*Don*	Present study, [12]	LT	SU	*Don* means “wide and short”; it is named so because its tuber does not elongate like other kinds. Tubers of *Don* varieties are similar in size and shape, but *Don babu* (fat man) has more strongly pigmented tubers and stems. Few farmers also explained that *Don bayye* (fat woman) show a tendency to branching
	*Don bayye*			SB	
	*Don babu*				
12	*Dizzu-Kachi*	Present study, [12]	LT	PU	*Dizzu* is the Sheko term for their Bench neighbors to the east and is said to come from Bench areas. If the tuber and basal stems of this variety has dark purple color, it is black (*tsaa’nseb*), whereas *ga’nseb* does not.
	*Dizzu-Kachi tsaa’nseb*			PB	
	*Dizzu-Kachi ga’nseb*				
13	*Kachi-kundi*^a^	[12]	LT	SB	*Kundi* means “feather or filament at rear end of a chicken” Tubers has a considerable numbers of spiny roots on its crown
14	*Lekut*	Present study	LT	SU	Tuber is white with purple at distal end and white elsewhere
15	*Logit*	Present study	LT	SU	Tuber is purple with white at middle and white elsewhere
16	*Shure*	Present study	LT	SU	*Shure* means “worm”. It is so named because tubers of this yam have a worm-like shape.
17	*Kachi-Kuch’ai*^a^	[12]	LT	SB	Tubers of easy cooking and tasty
18	*Konkay*	Present study	LT	SU	Name means “crispy while eating”. The tuber and basal stems of this variety has a pronounced dark purple color.
19	*Banda boy*	Present study, [14]	LT	SB	Name means “of multicolored”. White purple tuber and cylindrical shape. If it is white at distal end and white purple elsewhere, it is *Tsam*; if it is pale purple at proximal end and white elsewhere, it is *Tsenah*
	*Tsam banda boy*			PB	
	*Tsenah banda boy*				
20	*Shapinsin*	Present study, [14]	LT	SU	Tuber is flat, white at distal end, and purple white elsewhere
21	*Kuchuu’bai*	Present study, [14]	LT	SU	White tuber and irregular shape.
22	*Zansul*	Present study, [14]	LT	SU	Whole tuber is white and flattened at distal end
23	*Tsid boy*	Present study, [14, 15]	LT	SB	Whole tuber is dark purple
24	*Dal boy*	Present study, [14]	LT	SB	Whole tuber is white
25	*Don boy*^a^	[14]	LT	SB	Whole tuber is pale purple
26	*Shamut*	Present study, [14]	LT	SU	Irregular tuber shape.
27	*Kappar*	Present study, [14]	LT	SU	White purple tuber, very firm flesh, oval tuber shape.
28	*Kalu*^a^	[14]	LT	SU	White purple tuber and cylindrical shape
29	*Bud boy*^a^	[14]	LT	SB	Whole tuber is white with purple and oval shape.
30	*Tush boy*^a^	[14]	LT	SB	White purple tuber, very firm flesh, oval shape
31	*Bunkri*^a^	[14]	LT	SU	Tuber shape is in between cylindrical and oval
**II** ***Dioscorea alata***					
1	*Ongubay*, *Baday*	Present study, [12, 14, 15]	LT	SU	*Ongubay* means “foolish” while *Bada* means “distinct”. It is so named because its foliage differs from that of other kinds of domestic *Kachi*. Whole tuber is white, but if its tuber and stem edges has dark orange color, it is red (*submseb*)
	*Submseb Ongubay*	Present study		SB	
2	*Earkubai*	Present study, [12, 14, 15]	LT	SU	Name means “of sharp taste”. Tuber and stem edges of this variety have purple pigment and irregular in shape.
3	*Zenkuru*	Present study, [12, 15]	LT	SU	Name means “seeker of others”. It is so named from a lazy man who does not work but seeks to eat food that is prepared by another person
4	*Dak’oi*^a^	[12]	LT	SU	Whole tuber is white and irregular tuber shape.
**III** ***Dioscorea bulbifera***					
1	*Karka-ama* or *Balakay-oake*	Present study, [12, 14]	W	PU	Classification of a *bulbifera* plant as wild vs. cultivated relied on the area of growth. *Ama/oake* grows in garden while *Karka ama/Balakay oake* grows in wild area. If the bulbils of cultivated *bulbifera* have traces of dark color near edges, it is black (*tsaa’nseb/Tiab*); if not, it is white (*ga’nseb/don*)
2	*Ama* or *Oake*	Present study, [12, 14]	LT	SU	
	*Ama tsaa’nseb/Tiab oake*			SB	
	*Ama ga’nseb/Don oake*				

Contexts: *W* wild, *WT* wild transplant, *RT* varieties of cultivated yams that are known to be recently transplanted to open field, *LT* varieties of cultivated yams that are regarded as longtime cultivars

Structures: *SU* simple unitary, *SB* simple binary, *PU* productive unitary, *PB* productive binary

^a^Indicates the list of names of varieties, where their characteristics are given based upon only on the folk-provided descriptors the fact that they were not encountered during the survey

**Table 3:** Sheko and Bench intermediate folk categories of yams at the sub-generic level

Folk basis	Categories	Folk labels/names	Characteristics of each category as described by local farmers	No. of varieties and sub-varieties in each category
Context	Wild	*Karka-ama* or *Balakay-Oake*	None-edible tuber	1 (0)
		*Karka-Kachi*, *Shay-boy*, *or Karckabat*	Very spiny vines and roots, vigorous, late maturing, flowering, seed producing, and tubers are thin elongated and bitter in taste.	3 (3)
	Wild transplant	*Karka-Kachi*, *Yasind*	Is very similar with wild yam, but its tuber tends to change to fat and tasty over the course of years of cultivation	2 (5)
	Cultivated	Recent transplants (I) + longtime variety (II)		
	I	*Kaibab-Kachi*	Medium to high spines on vine and roots, medium-sized light green leave, vigorous. Broad and tasty tuber.	4 (4)
	II	*Kachi/Boyye*	Few to medium spines on vine and tuber, medium-sized dark leaves, early maturing and flowering	22 (6)
		*Baday-kachi*	Non-woody, spineless and large sized green leaves. Early maturing and not flowering	4 (1)
		*Ama/Oake*^a^	Edible tuber	1 (2)
Gender	Female	*Kachi/Baday-kachi*, *Ama/Oake*^a^	Early maturing, less vigorous, double harvest, susceptible to stress, tasty tuber	24 (7)
	Male	*Karka-Kachi*, *Shay-boy*, *Yasind*, *Kaibab-kachi*	Late maturing, single harvest, vigorous, stress tolerant, bitter taste tuber	9 (12)

^a^ Refers to aerial yam, values in parenthesis are no. of sub-varieties (*N* = 21); values without parenthesis are the No. of varieties (*N* = 37)

### Ethnolinguistic analysis of folk names

#### Meaning and origin of names

Names of yam varieties are derived from a wide range of sources. The sources from which names are originated include locations where it grows or comes from, attributes of a persons, or social groups, name of animals or pests, plant morphological characteristics, and others. Translation of names revealed that 49% of the names have meanings while the rest have no meanings, or their word origin is lost. The lists of names and their implied meanings are presented in Table 2. Naming is based on a consistent set of linguistic categories. A survey with 15 fixed landrace samples showed that about 88% of the landraces are consistently named across locations. Naming was considered consistent if more than 80% of the seed lots to which a particular name was attached represented the same variety [32]. Identification and distinction of the taxa assigned to different categories is also made based on a consistent set of characters and character states. Throughout the region, names and naming of varieties and sub-varieties of yams are often relying on specific plant traits.

### Structures of names

#### Structures of variety names

Naming of varieties takes place in two structures: those that are composed of a single word (unitary names), and those that are consisted of two words (binary names). Unitary names can be divided into two obvious classes. Some unitary names such as *Chabsha* and *Yasind* are linguistically unanalyzable in form, containing unique words which can be shown to be semantically unitary and linguistically distinct. These expressions are known as simple unitary names. Others such as *Karka-Kachi*, *Shah-boy*, and *Kaibab-kachi* are analyzable linguistically. They are noticeable in that their expression shows the origin of a subordinate category. For instance, *Karka-Kachi* is kind of wild *Kachi* obtained from wild area, and *Kaibab-kachi* is a kind of domestic *Kachi* originated from *Kaibab* area, and so on. These forms are known as productive unitary names. Few yam varieties are also labeled with binary structures. Binary forms, like productive unitary forms, are identifiable in that each of the expression carries a modifier that marks a characteristic of the subordinate category. But, binary forms differ from productive unitary forms in that the modifiers they carry are related to specific plant characteristics. These forms are known as simple binary names. Examples of simple binary names include *Tsid boy* and *Dal boy*. Over 78% of the varieties are labeled with unitary names whereas the rest are binary names, indicating that the labeling system used for naming a given variety is skewed to uninominal (Table 2).

#### Structures of sub-variety names

Examination of the naming structure used for labeling sub-varieties shows that all consisted of binary names (Table 2). Two forms of binary names can be recognized. One group contains simple unitary names of a given variety in a modified form such as *Tsid Chabsha* and *Dal yasind* are noticeable in that the modifiers they carry are related with specific characteristics of a sub-variety. These forms are known as simple binary names. Other groups such as *Karka-kachi submseb* and *Kaibab-kachi ga’nseb* are identifiable in that they carry modifiers of a variety plus modifiers related with the characteristics of a sub-variety. These forms are known as productive binary names. The basic name of a sub-variety is often a binomial structure consisting of a word designating the variety plus a prefix/suffix added to it. The prefix/suffix is meaningless alone because several sub-varieties in different varieties may have the same specific prefix/suffix (Table 2).

### Folk descriptors

#### Identification of varieties and sub-varieties

A total of 37 named varieties are recognized by farmers as distinct. The lists of names of varieties together with descriptions of the characters that the farmers used to distinguish them are presented in Table 2. Farmers used a total of 14 characters and 43 character states for distinction and identification of varietal categories (Table 4). More than half of these refer to aspects of a variety’s morphology, thus showing that morphological characters played a key role in the local distinction of yam varieties. Of these, tuber characteristics such as shape, color, size, and texture played a key role in the local identification of varieties. In some cases, non-tuber traits such as stem or leaf characters are also used. Non-plant characters such as location where it grows or comes from and attributes of persons or social groups can also be used to distinguish between varieties. For example, classification of a *bulbifera* variety does not depend on the physical traits of a plant but of its location. Farmers described that *Ama*/*oake* grows in gardens, whereas *Karka-ama*/*Balakay-oake* grows in wild places (Table 2).

**Table 4:** Comparison of Sheko and Bench folk classification of yams with the formal divisions

Scientific ranks	Formally recognized ranks	Ranks recognized by Sheko and Bench farmers	Number of taxa identified in each ranks	No. of descriptors used to classify and name the taxa assigned to each ranks	
				Characters	Character states
Family	Life form	-	-	-	-
Genus	Generic	Folk generic	4 individual labels	1	2
Species	Sub-generic	Supra-variety	9 labeled groups	11 (19)	29 (54)
Variety	Varietal	Variety	37 individual labels	5 (14)	14 (43)
-	Sub-varietal	Sub-variety	21 individual labels	1	4
**Overlapping descriptors**					
1	Between generic and supra-varietal ranks			1	2
2	Between generic and varietal ranks			1	2
3	Between supra-varietal and varietal ranks			7	23
4	Between varietal and sub-varietal ranks			1	4
**Summary of overall descriptors**				**26**	**74**

*NB* numbers in the parenthesis refers to the total number of characters and character states of the respective ranks

The lowest level of yam taxonomy is the sub-variety, i.e., composed of further divisions of a given variety. The lists of names of sub-varieties together with the description of the descriptors that the farmers used to distinguish sub-varieties are presented in Table 2. Altogether, 21 sub-varieties are recognized by farmers as distinct. Some sub-varieties of a given variety were reported to have the indicative features of the main variety; each was also reported to differ from all the others and the main variety at least in one aspect. Distinction and identification of sub-varietal category is principally or sometimes exclusively relying on the color of the tuber.

#### Supra-variety categories

Farmers have a number of additional systems for grouping yam varieties. Two major ways of groupings can be identified. One grouping distinguishes yams by cultivation contexts, describing them as cultivated, wild, or wild transplant. A second grouping distinguishes yams by gender describing them as female or male. Each of these groupings is formed by assembling several varieties and sub-varieties together; they are thus supra-variety categories, i.e., are groupings higher than the folk variety. Farmers used a total of 19 characters and 54 character states while grouping of yams by contexts and gender (Tables 3 and 4). The characteristics of the different supra-variety categories and the underlying basis of the classification are presented below.

#### Groupings on the basis of contexts

On the basis of contexts, yam varieties fall into three main supra-variety groups. These are (a) yams of the *D. cayenensis* complex or *D. bulbifera* that are growing wild; (b) yams of the *D. cayenensis* complex that have been recently transplanted from wild location to garden; and (c) yams of the *D. cayenensis* complex, *D. bulbifera*, or *D. alata* that are of under cultivation.

##### Wild-growing yams

Wild yams are morphologically related to some domestic yams, but unlike domestic yams, they have not been domesticated yet and exist in wild contexts. The term “wild” here refers to plants that are growing wild in the forest and had no known history of human manipulations. It also assembles volunteer plants growing in uncultivated areas without farmers’ help. In the studied area, wild growing yams are found in degraded forests, field margins, river banks, and disturbed habitats. Such yam types are known in Sheko as *Karka-Kachi* as a general category, with different one word names for smaller categories. In Bench, such yam types are known as *Shah-boy* as a general category, with no smaller categories (Table 2). The folk basis and the characteristics of this supra-variety category are presented in Table 3.

##### Wild transplant yams

Wild transplant yams are those yams transplanted from wild contexts and grown in home garden beneath a large tree. Unlike the cultivated yams that are replanted in an annual cycle, yams in this supra-variety group are left in the same place for many years. Hence, this group of yam qualifies as yams under domestication and not just as yams under cultivation. We therefore designate this supra-variety group as “wild transplants” to differentiate it from wild and cultivated yams. Across the study area, many gardens contain a few yams that seem on the state of transition, yams transplanted from wild contexts where many of them had no known history of cultivation in an open field along row of stakes. In Sheko, such yams are usually known as *Karka-kachi* while it is called *Yasind* in Bench as a general category, with each of them has different one word names for smaller categories (Table 2). The folk labels and the characteristics of this supra-variety category are presented in Table 3.

Farmers did not provide consistent morphological grounds for differentiating between wild and wild transplant yams. Yet, some farmers are able to describe the distinction between the two contexts. According to farmers, wild transplant yam retains its wild traits for the first few years after transplantation, and thus, they knew it by the name of wild places though it grows in a home garden. It begins to take on the traits of domestic yam, and in fact, farmers credit wild transplant yams as having broader and tasty tubers over the course of 3 to 5 years of cultivation. If they are satisfied with the modifications, they may rename it to a variety of domestic yam it resembles most closely. Sheko usually renames *Karka-Kachi* as *Torbay* or *Kaibab-Kachi*, whereas the Bench renames *Yasind* as *Chabsha* or *Tolubab*. There is no strict rule in naming, and renaming to a variety of other domestic yam is also possible.

##### Cultivated yams

This group of yams is further divided into two main supra-variety categories: those known to be recent transplants and those regarded as longtime variety.

##### Those known to be recent transplants

This group represented a variety of cultivated yams, but their morphotype is closely related with wild transplant yams. Some individual plants are known to be recently transplanted to open field. Farmers reported four varieties that belong to this supra-variety category, namely *Kaibab-kachi*, *Chabsha*, *Torbay*, and *Tolubab* (Table 2)*.* Some of these names can also be used interchangeably for yams that have been recently transplanted from wild contexts and grow beneath a large tree. The name *Kaibab-Kachi* is sometimes used as a gloss for all varieties in this supra-variety category (Table 3).

##### Those regarded as longtime varieties

Of all yam varieties recorded, 27 have a longtime history of cultivation by humans (Table 2). They are usually grown in small plots of open field along rows of stakes. This group of yams can be further divided into three obvious classes. The first class assembles 22 varieties, most of which seem to have similarity to the *D. cayenensis* complex (Table 2). Of all the yam species, this complex is the most economically important, but also has the greatest number of locally threatened varieties. Six varieties, namely *Shure*, *Don*, *Kappar*, *Dal boy*, *Zansul*, and *Kuchuu’bai* are undergoing erosion. Others such as *Kachi-kundi*, *Kachi-Kuch’ai*, *Kalu*, *Bud boy*, *Tush boy*, and *Bunkri* are already abandoned. Farmers usually labeled them by the name *Kachi/Boyye* (Table 3). Despite the scientific taxa that describe their origins to separate areas in Africa, farmers regarded all of them as native to their area.

A second class comprises four longtime varieties belonging to *D. alata* (Table 2). Of these, *Baday* is the most common, both in terms of its distribution and relative abundances. Farmers usually labeled them by the name *Baday-Kachi* (Table 3). The names *Baday* and *Ongubay* are used interchangeably for one another and sometimes used as a gloss for all varieties of *D. alata*. This species is not native to Ethiopia, and in Ethiopia, it grows only in cultivated forms [11]. Farmers readily distinguished a variety of *D. alata* due to its large-sized foliages and four-winged stems. *D. alata* varieties had no known history of introduction to these areas, but farmers regarded all of these varieties as native to their area. *D. alata*, thus, must have first entered these areas as a cultivar well before the time periods known in local oral historic memory [12]. The meaning of the names of *Baday*, one of the most common variety (Table 2), also confirm the speculation made by Hildebrand [12] and suggests that farmers are aware of the distinct nature of this yam in relation to other kinds of domestic yam.

A third group comprises a single cultivar of *bulbifera* that is labeled by the name *Ama-/Oake*. It has two sub-varietal categories. Distinction between the two categories is relied on traces of dark pigmentation on the portions of the bulbils. The *bulbifera* plant also occurs in wild form. Farmers did not provide consistent morphological grounds for discriminating between the two contexts. Identification of a *bulbifera* variety as wild vs. cultivated does not depend on the physical traits of the plant but of its location and edibility of the bulbils (Tables 2 and 3).

#### Groupings on the basis of gender

On the basis of gender, yam varieties fall into two supra-variety groups: female (*Mine/Baye*) and male (*Babu/Eyane*). The distinction of varieties as male or female is not related to the biological reproduction of varieties. Farmers’ distinction most of the time depends on maturity stage, time of harvest, growth habit, taste of the tubers, and tolerance to stress. Some farmer claims early maturing, less vigorous, double harvest, and tasty varieties as female, while they are male otherwise. For instance, late maturing domestic yams such as *Chabsha*, *Torbay*, and *Tolubab* are among others that farmers considered as a male variety. All the wild and wild transplant yams were also considered as male varieties. Others such as *Dizzu-Kachi* and *Tsid boy* are among others that are recognized as female varieties (Tables 2 and 3).

### Comparisons between folk and formal taxonomy

Sheko and Bench classification and naming of yam allows for the recognition of four distinct folk ranks. Each of these can be considered as belonging to different levels and thus arranged hierarchically. In order from the least to the most inclusive group, the categories are sub-variety, variety, supra-variety, and folk generic. Table 4 provides comparison of formal and folk botanic systems. Applying Berlin et al. [26] and Berlin [31] schemes, the folk-recognized ranks can be assigned to four formal biological divisions such as sub-varietal, varietal, sub-generic, and generic. Taxa assigned to the folk generic and varietal categories correspond to the formally recognized divisions at the genus and varietal levels, respectively. Taxa assigned to supra-variety category may also correspond to formally recognized ranks at the sub-generic level.

One interesting observation between the two system is that formal taxonomy focuses at and above species level, while folk taxonomy concentrates on intraspecific diversity, i.e., at the folk varietal and sub-varietal ranks. We found the highest number of taxa at the folk varietal and sub-varietal ranks. The recognition of rich taxa at the lowest folk ranks can be taken as a clear evidence for the existence of a moderate overlap between the two systems. Yet, the overlap is far from perfect, and the standards in folk taxonomy are not expected to be absolutely consistent across locations and different social domains; there are thus some anomalies. In view of this, the two systems can be combined in order to obtain insights into the links of classification rationale and systems applied to the management and utilization of on farm genetic resource.

## Discussion

### Indigenous biosystematics of yam

The folk taxonomic systems reported in Sheko and Bench show a pattern of hierarchy starting from the least to the most inclusive groups. Farmers recognize at least 58 individual taxa (Table 2). This suggests that Sheko and Bench taxonomy of yam is over-differentiated at the intraspecific level, i.e., at varietal and sub-varietal ranks. Such over-differentiation is rare and is often related with crops of great importance [26, 30, 31]. A similar distinction has been made to yam in South and Southwestern Ethiopia [12–14]. The same trend of using folk biosystematics was reported for enset in Southern Ethiopia [33, 34] and for sorghum in Eastern Ethiopia [35].

We also found nine supra-variety categories that are labeled by names (Table 3). Applying Berlin [31] scheme, these categories constitute mid-level ranking rather than separate taxa. The recognition of labeled intermediate taxa is highly relevant in order to obtain insights into the basic principles of native taxonomy and should not be ignored by placing too much stress solely on the named groups at smaller ranks. It is rare in most formal taxonomic system, and only a few works has yet reported such taxa. Such ranks have been reported in folk taxonomy of potatoes by the Quechua communities in the Andes [17, 18]. At and above species level, Sheko and Bench recognize four generic taxa (Table 1). Growing location was used as the main differentiating criterion for recognizing and naming of yams at the generic level. Our result is in accord with other reports on yam in Ethiopia [12] and in Oceania [36, 37], enset in Ethiopia [34], and species of Mesoamerican columnar cacti [3, 38] and potato in the Andes [17–19], where the local farmers in each of these areas recognize the genus level folk taxa in a similar way.

A basic ethnolinguistic analysis of folk names unraveled the meaning, origin, and structure of naming categories applied to yams. The meaning of the names is quite descriptive of the plant characteristics or its geographic origin. Besides, knowledge of the names and the characteristics of locally recognized taxa is widely shared within the community and across locations. This is evident in the recognition of a consistent set of folk names across locations as well as in the use of a consistent set of characters and character states for recognizing the different taxa. This suggests that there appears to be strong relationships between the ethnolinguistic forms of naming and the characters with which it labels. But, for the majority of the recorded names, their implied etymology was not explained by farmers. In Ethiopia, similar unexplained names were reported in these and other areas for yam and enset varieties [12, 14, 33, 34]. Examination of the labeling systems revealed that they are of two types structurally. Unitary structure was found to be predominant in variety names while all the sub-varieties were labeled with binary names. The existence of two distinct structures at the lowest botanic levels can be taken as a clear evidence of the existence of two separate folk groups. Studies from Ethiopia [12, 25, 33] reported a similar labeling system for varieties and sub-varieties of yam and enset.

Farmers used a wide range of folk descriptors for identification of taxa at varietal and sub-varietal ranks (Table 2). The lowest level of folk rank is sub-variety, where the primary contrast is between tuber colors. The folk descriptors identified in this study are comparable with descriptors reported for yam in Ethiopia [12] and in Oceania [37], where farmers relied on tuber color for sub-varietal distinction. Folk descriptor recognized in our study is also comparable with that reported for potato in the Andes [16–18, 39], where the only distinction between sub-varieties of potato is color of the tubers. The determination of a variety’s identity is made by considering a number of specific plant characteristics. Of these, variety is mainly distinguished by the shape, color, and size of the tubers. Non-tuber characters such as location, attributes of persons, or social groups are also used to distinguish yam varieties. The folk descriptors recorded in this study are comparable with those reported for yam in Ethiopia [12, 13, 25]. Folk descriptors recognized for yam also share the descriptors reported for enset in Sidama and Wolaita [33, 34].

Analysis of the ethnobotanical data showed that the individual units are further grouped into a number of named categories. Sheko and Bench recognize three main supra-variety categories by contexts, describing them as cultivated, wild, or wild transplant. Four additional subordinate groups are recognized within the cultivated groups, bringing the total number of labeled groups to seven. Each of these categories is made up of small subpopulations that are morphologically distinct, yet share some overlapping character states that the farmers used to group them (Table 3). This reflects the diversity forms of cultivated yams through which farmers drive its evolution under domestication. A similar distinction has been made in Ethiopia with yam [12]. Sheko and Bench also recognize two main supra-variety groups of yam on the basis of gender, describing them as male or female. Gender can thus be considered as a distinct way of classifying supra-variety groups and, hence, influence practical decisions of maintaining diversity. Gender-based classification system was reported for yam in Ethiopia [14, 25]. This gender-related classification is not limited to yam, where similar studies [33, 34, 40] reported gender-based classification of enset, showing that different social groups in Ethiopia classify crop plants in a similar way.

### Comparison between formal and folk taxonomy

The local classification system allows for the recognition of a folk biological category consisting of four ranks (Table 4). Sheko and Bench taxonomy of yam shares the general classification schemes reported for enset in Ethiopia [33], potato and cassava in the Andes [16, 17, 39, 41, 42], columnar cacti in Mexico [38], and folk botanical classification of plants in Switzerland [43]. A closer look of the two systems shows a moderate overlap, the most frequent being difference in scales. In the formal taxonomy, varietal taxa occur in contrast sets of few members, while in folk taxonomy it occurs in contrast sets of many members. Contrast sets of more than two members tend to refer to plants of major cultural importance [26]. Though there is a modest overlap, they are arguably complementary at least for two reasons. First and more notably, the folk-recognized taxa are consistent to a considerable extent with the variability obtained with standard markers (Tsegaye B, Bizuayehu T, Wendawek A: Assessing Morphological Diversity in Ethiopian Yams (Dioscorea spp.) and its Correspondence with Folk Taxonomy, forthcoming), confirming earlier reports from Ethiopia [25, 27, 28, 44] and West Africa [45–47]. This suggests that biological and functional consideration constitutes the basis of folk taxonomy. Second, farmers manage a wide range of descriptors, each of which contains several character states with inherent labeling systems. This provides additional support to the view that plant classification is not an activity confined to the domain of formal science only.

## Conclusion

A number of conclusions can be drawn from the study. First, analysis of the local classification systems in Southwest Ethiopia suggests that within the province where cultivation of yam is feasible, Sheko and Bench botany of yam is unique. It is polytypic in Sheko, as is also in Bench. This is evident in the recognition of four taxonomic ranks. Second, farmers used a wide range of descriptors for recognizing the different taxa, most of which correspond to the formal descriptor list. As a result, the two systems can be treated as complementary, and conservation efforts should take both systems into account.

Third, farmers recognize various individual taxa. Each of these is perceived as distinct and given a separate name. The recognition of rich intraspecific taxa is *prima facie* evidence for great diversity and can be used as the unit of on farm diversity. Individual households are the primary management unit of selection and maintaining diversity. Assessment of diversity kept by diverse households is essential to obtain insights into the nature and spatial dynamics of diversity, and this assessment can rely on folk-recognized taxa. Research on diversity can draw directly on folk biosystematics as long as the unit of analysis is the household.

More generally, this study documented folk biosystematics of yams by the far Southwest Ethiopians, constitutes a complex of dynamic indigenous knowledge that is highly relevant for conservation efforts. Yet, the study is not exhaustive; no objective generalizations can thus be drawn regarding the actual extent of diversity in Ethiopia. The problem is further complicated by the fact that for the majority of the recorded names, their etymons and implied descriptions are incomplete. Thus, more detailed ethnobotanical studies in these and other areas are of paramount importance to unravel such difficulties.

## Data Availability

The ethnobotanical data gathered during the current study are included in the manuscript. Other supplementary materials such as photographs and field notes of varieties are available by request to the authors.
